# Development and External Validation of a Prognostic Nomogram for Metastatic Uveal Melanoma

**DOI:** 10.1371/journal.pone.0120181

**Published:** 2015-03-17

**Authors:** Sara Valpione, Justin C. Moser, Raffaele Parrozzani, Marco Bazzi, Aaron S. Mansfield, Simone Mocellin, Jacopo Pigozzo, Edoardo Midena, Svetomir N. Markovic, Camillo Aliberti, Luca G. Campana, Vanna Chiarion-Sileni

**Affiliations:** 1 Melanoma Oncology Unit, Veneto Region Oncology Research Institute (IOV-IRCCS), Padova, Italy; 2 Department of Surgery, Oncology and Gastroenterology, Padova, Italy; 3 Department of Internal Medicine, Mayo Clinic, Rochester, Minnesota, US; 4 GB Bietti Foundation-IRCCS, Rome, Italy; 5 Department of Statistical Sciences, University of Padova, Padova, Italy; 6 Division of Medical Oncology, Mayo Clinic, Rochester, Minnesota, United States of America; 7 Department of Ophthalmology, University of Padova, Padova, Italy; 8 Interventional Radiology, Veneto Region Oncology Research Institute (IOV-IRCCS) Padova, Italy; 9 Sarcoma and Melanoma Unit, Veneto Region Oncology Research Institute (IOV-IRCCS) Padova, Italy; University of Tennessee, UNITED STATES

## Abstract

**Background:**

Approximately 50% of patients with uveal melanoma (UM) will develop metastatic disease, usually involving the liver. The outcome of metastatic UM (mUM) is generally poor and no standard therapy has been established. Additionally, clinicians lack a validated prognostic tool to evaluate these patients. The aim of this work was to develop a reliable prognostic nomogram for clinicians.

**Patients and Methods:**

Two cohorts of mUM patients, from Veneto Oncology Institute (IOV) (N=152) and Mayo Clinic (MC) (N=102), were analyzed to develop and externally validate, a prognostic nomogram.

**Results:**

The median survival of mUM was 17.2 months in the IOV cohort and 19.7 in the MC cohort. Percentage of liver involvement (HR 1.6), elevated levels of serum LDH (HR 1.6), and a WHO performance status=1 (HR 1.5) or 2–3 (HR 4.6) were associated with worse prognosis. Longer disease-free interval from diagnosis of UM to that of mUM conferred a survival advantage (HR 0.9). The nomogram had a concordance probability of 0.75 (SE .006) in the development dataset (IOV), and 0.80 (SE .009) in the external validation (MC). Nomogram predictions were well calibrated.

**Conclusions:**

The nomogram, which includes percentage of liver involvement, LDH levels, WHO performance status and disease free-interval accurately predicts the prognosis of mUM and could be useful for decision-making and risk stratification for clinical trials.

## Introduction

Uveal melanoma (UM) is the most common primary intraocular malignancy in the adult, representing 5–6% of all melanomas (annual incidence in Europe approximately 5:1000000), and is associated with age and, light skin and blue pigmented eyes[1,2]. Although local control is achieved in most cases, approximately 50% of patients will develop systemic disease [1]. Although the liver is the most common site of metastatic disease, UM can metastasize to any including the lungs, bones, soft tissues, gastrointestinal tract, ovaries, kidneys and central nervous system (CNS)[2, 3]. The reported median life expectancy of patients affected by metastatic UM (mUM) ranges from 3.6 to 15 months [4]. Site; number and diameter of metastases; percentage of liver substitution, presence of symptoms; alteration of liver function tests, especially alkaline phosphatase (ALP) and lactic dehydrogenase (LDH); older age; male sex; and a shorter metastasis-free interval have been associated with a poorer prognosis [2, 5–10]. Due to several known and unknown factors, as for mucosal, acral and skin melanomas, UM is a poor responder to antiblastic chemotherapy and radiotherapy[11]. Moreover, UM cannot benefit from target therapy tailored for cutaneous melanoma, as BRAF inhibitors, because of the absence of the target mutation[12] and, for reasons yet to be explored, response to new immunotherapies is poorer than for cutaneous melanoma[13]. Treatments for mUM can be divided into liver directed treatments; such as surgical resection [9], ablation [14], radiation [14], hepatic arterial chemoinfusion [15, 16], immunoembolization [17], transarterial chemoembolization [18], radioembolization [19], isolated or percutaneous hepatic perfusion [20, 21]; and into systemic treatments; such as chemotherapy (antineoplastic drugs used alone or in combinations [22]), immunotherapy (interferon [23], interleukin-2 [24], and, more recently, ipilimumab[13]), anti-angiogenetic drugs [25, 26], and targeted agents such as MEK inhibitors [27, 28]. Despite the efforts to improve mUM outcomes, prognosis remains poor and clinicians lack a standard prognostic tool. The study objective was to identify the independent prognostic factors for mUM in order to formulate a reproducible prognostic algorithm, that could be easily enough to be integrated into clinical practice.

## Patients and Methods

### Patients and Therapies

The prospective melanoma databases at the Melanoma Oncology Unit of the Veneto Oncology Institute (IOV) and at Mayo Clinic, Rochester (MC) were queried under institutional review board approval for mUM. IOV patients (N = 152) were diagnosed and treated between September 1990 to October 2013, MC patients (N = 102) were diagnosed and treated between January 2000 and August 2013. The majority of patients from IOV (72.4%) and MC (84.3%) were treated with Iodine-125-brachitherapy for their primary melanoma; the remaining patients received enucleation, with the exception of those in whom treatment of their primary melanoma was futile as they presented with stage IV disease. Material for fluorescent in-situ hybridization analysis (FISH) of the primary tumor was obtained with a 25-gauge trans-scleral fine needle aspiration biopsy. FISH was used to evaluate the cell karyotype and alterations of chromosomes 1, 3, 6, 8 and 10. Procedure and testing was performed using previously published methods [29–31]. Metastases were discovered at initial staging (6 IOV patients and 3 MC patients) or during follow-up via ultrasound tomography or computed tomography (CT). Diagnosis was confirmed via core biopsy or fine needle aspiration cytology. Staging was completed with CT or magnetic resonance (MR) when not previously performed. Therapy was chosen according to the localization of metastases and the availability of clinical trials.

Gender, age; date, size and characteristics of UM; date and site of metastases; date, type and outcome of therapies; date of last follow up or death and cause of death were collected from patient records. Date and cause of death were collected from local registry offices, and telephone interviewing of family or from general practitioners for patients lost to follow up. Levels of LDH, alkaline phosphatase (ALP), ϒ-glutamyltranspeptidase (ϒGT) and transaminases were recorded at diagnosis of metastatic disease and were computed as proportion of the respective upper normal value. The site and extent of metastases at baseline were quantified with CT (IOV cohort and most MC patients) or MR (a minority of MC patients). Liver metastasis volumes were calculated with three-dimensional reconstruction by helical CT or MR of the liver and registered as the percentage of liver substitution; we retrospectively reviewed CT images of the IOV cohort with Syngo CT Oncology software (version 2009E, Siemens, Germany) for confirmation and to assess the maximum diameter and the number of liver metastases. It was decided not to include this information as the percentage of liver substitution was considered the best indicator of effective volume of hepatic disease. Moreover, some patients had many small metastases, making analysis of diameter and number very complex.

The study was approved by the Institutional Review Boards of Veneto Region Oncology Research Institute, Padova, and Mayo Clinic, Rochester. All clinical investigations have been conducted according to the principles expressed in the Declaration of Helsinki. All patients gave written informed consent to the use of their records for research purposes.

### Statistical Analysis

Patient features and clinical characteristics were analyzed using the Mann-Whitney two-tailed U test (continuous variables) and χ^2^ test (categorical variables). Disease-free interval (DFI) was defined as the time from initial UM diagnosis to first noted metastasis. Overall survival (OS) was defined as the time from diagnosis of first metastasis to the date of death or last follow-up. OS was estimated using the Kaplan-Meier method and survival between cohorts was analyzed using the log-rank test. Cox proportional hazards regression was used on the IOV dataset to examine the association between potential prognostic variables and survival. Age at diagnosis of UM was not tested because it collimated with the other temporal covariates. Schoenfeld residual-based methodology was used to verify the proportional hazard assumption of the Cox model. The Wald test was used to assess the significance of each variable included within the full model. Only variables with *p* values ≤.05 were maintained in the final model after fast-backward variable selection. The performance of the model was measured in terms of calibration (with the area under the receiver operating characteristic curve) and discrimination (Harrell’s C-index). Shrinkage slope after 100 bootstrap replications was calculated as a measure of overfitting. The prognostic model was externally validated using the MC dataset. The prognostic nomogram was tailored using the final regression model with the total number of points derived by specifying values used to calculate the expected survival probabilities at 6, 12 and 24 months. Missing values were estimated with multiple imputation using additive regression, bootstrapping, and predictive matching. The estimation procedure was corrected using 20 multiple imputations. Patients whose death was unrelated to UM progression were censored at last follow-up (2 IOV and 0 MC patients). *P* values were calculated using two-tailored testing, and confidence intervals (CI) are reported at the 95% level. Statistical analysis was performed using R 2.15.2 (survival, Hmisc and rms libraries).

## Results

### Patient Characteristics

Descriptive statistics are summarized in Table 1.

**Table 1:** IOV and MC patient characteristic at diagnosis of stage IV disease.

Patients characteristics	IOV (*N* = 152)		MC(*N* = 102)		
	*N* (%)	Median (range)	*N* (%)	Median (range)	*P*
Sex					.271
Female	73 (48.1)		41 (40.2)		
Male	79 (51.9)		61 (59.8)		
Age of primary UM (years)		60.9 (25.3–82.5)		59.0 (28.0–92.0)	.352
Age of 1^st^ metastasis (years)		63.6 (34.3–89.4)		61.5 (30.0–92.0)	.262
DFI (months)		25.2 (0–339.2)		24.1 (0.1–140.8)	.703
Number of organs involved					.803
1	108 (71.1)		72 (70.6)		
2	32 (21.0)		19 (18.6)		
3≤	12 (7.9)		11 (10.8)		
Liver metastasis					**<.001**
No	9 (5.9)		14 (13.7)		
Yes	143 (94.0)		88 (86.3)		
<20%	63 (41.4)		68 (66.7)		
20≤50%	48 (31.5)		16 (15.7)		
50%≤	19 (12.5)		4 (3.9)		
*Missing data*	14 (9.2)		0		
LDH (x UNL)		0.8 (0.3–15.6)		0.9 (0.5–10.4)	**.005**
ϒGT (x UNL)		1.1 (0.1–27.9)		0.9 (0.4–25.3)	.761
AST (x UNL)		0.7 (0.2–11.8)		0.6 (0.3–10.4)	.901
ALT (x UNL)		0.6 (0.2–7.6)		0.9 (0.3–3.1)	.109
PS					**.008**
0	93 (61.1)		61 (59.8)		
1	46 (30.3)		9 (8.8)		
2	9 (5.9)		2 (1.9)		
3	4 (2.6)		1 (0.9)		
*Missing data*	0		29 (28.4)		
1^st^ line treatment of mUM					**<.001**
No treatment	21 (13.8)		0		
Any treatment	131 (86.2)		102 (100)		
Systemic therapy alone	56 (54.9)		74 (72.5)		
Locoregional therapy alone	17 (16.7)		17 (16.7)		
Locoregional + systemic therapy	68 (44.7)		5 (4.9)		
*Missing data*	0		9 (8.8)		

IOV = Veneto Oncology Research Institute,

MC = Mayo Clinic,

PS = Performance Status (according to World Health Organization classification),

(x UNL) = multiples of the upper normal limit.

Significant differences in the clinical characteristics between the two cohorts were noted in the percentage of liver substitution (MC patients had a lower frequency of hepatic metastases, and a lower percentage of liver substitution) performance status (PS) (MC cohort had lower frequency of patients with worse PS) and median values of LDH (IOV cohort had a lower median value).

Out of the 152 patients in IOV series, 131 received at least 1 line of therapy within 2 months from diagnosis of stage IV disease while 21 patients did not receive any treatment due to poor PS (4 had World Health Organization PS 3) or patient preference. All patients from MC received treatment for mUM with a higher percentage receiving systemic therapies over locoregional or combined approaches (locoregional plus systemic).

After a median follow-up of 11.4 months (0.4–89.9), 33 (22%) out of 152 patients from IOV were alive and 11 (7.3%) were lost to follow-up. Among the 108 patients from IOV who were deceased at the time of the analysis, 107 (98.0%) died of liver failure due to disease progression, and one patient treated by IHP with melphalan died of acute liver failure 2 days after the procedure. The median follow-up of the 70 (68.6%) MC patients alive or lost to follow-up at the time of the analysis was 14.9 months (1.0–55.8), all deaths were due to disease progression.

### Survival and Prognostic Nomogram

**Fig 1:**
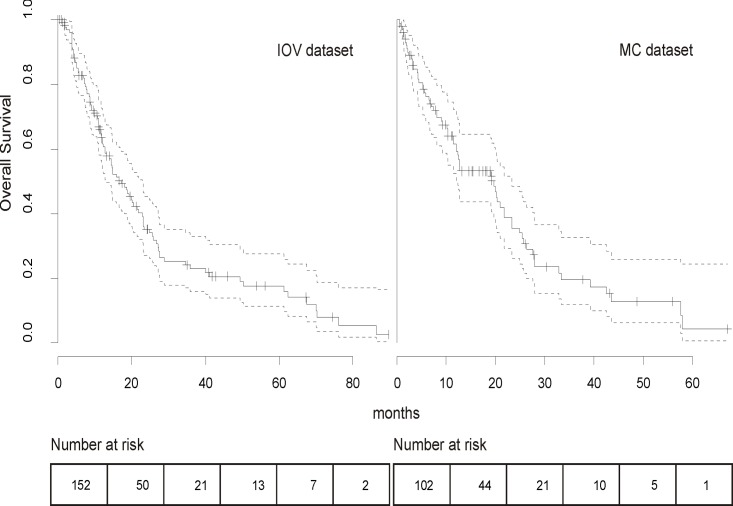
Overall survival of IOV and MC patients after diagnosis of first metastasis. No statistical difference was noted between the two survival curves (*P* = .271). Of note, both curves show a slope flatting after 20 months. Dotted lines refer to the 95% CI.

**Fig 2:**
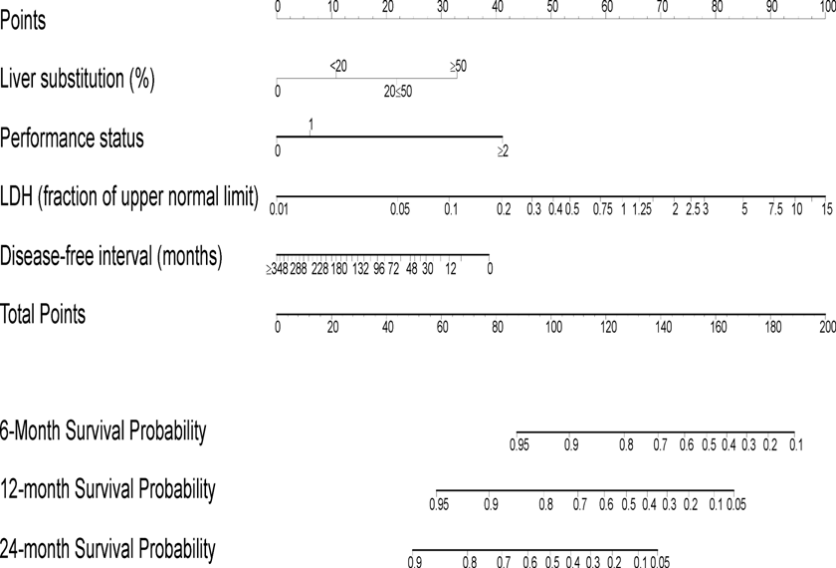
Nomogram of the final prognostic model. The sum of the prognostic factor points corresponds to the survival probability at 6, 12 and 24 months.

The estimated median OS was 17.2 months (range 1.2–86.4) and 19.7 months (range 12.4–23.4) in the IOV and MC series, respectively. Twelve and 24-months survival was 63.4% and 34.5% for IOV and 61.7% and 35.6% for MC patients, respectively (Fig. 1). The survival analysis demonstrated that the difference between the two cohorts was not significant (*P* = .271).The following covariates were tested in a multivariate Cox regression model: sex, size and characteristics of primary UM, age at diagnosis of mUM, DFI, hepatic enzyme levels at diagnosis of mUM (LDH, ALP, ϒGT, transaminases), site and number of metastases, percentage of liver replacement and first line treatment. We observed that locoregional therapy was associated with a trend for longer survival [32, 33], although this was not statistically significant in the multivariate model. Clinical and histopathological characteristics of primary melanoma did not correlate with survival. A trend for shorter DFI in patients with worse primary melanoma characteristics, i.e. stage (*P =* .060), ciliary body involvement (*P =* .058) and epitheloid (*P* = .069) or mixed histology (*P* = 0.57) was noted, however not statistically significant. We observed enrichment for strongly pigmented and genomically aberrant cases. S1 table shows the correlation with survival for primary melanoma characteristics and their influence on OS at multivariate analysis. The significant covariates of the final model are showed in Table 2 while the nomogram for the predictive model is reported in Fig. 2. Increasing liver substitution (HR 1.6), serum LDH (HR 1.6) and PS (HR of 1.5 and 4.6 for PS 1 and 2–3, respectively) were associated with worse OS. Longer DFI was associated with better prognosis (HR 0.9). The effect of each predictor on survival is represented in S1 Fig.

**Table 2:** Predictive factors used in the final prognostic model.

Prognostic factor	Median value (range)	*N* of patients (%)	HR	*P*
PS				**<.001**
0 as baseline with HR = 1		93 (61.1)	1	
1	-	46 (30.3)	1.5	
2–3	-	13 (8.5)	4.5	
LDH (x UNL)	0.8 (0.3–15.6)	-	1.6^a^	**.014**
Liver substitution (%)	20 (0–80)	-	1.6^a^	**<.001**
Disease-free-interval (months)	25.2 (0–339.2)	-	0.9^a^	**<.001**

PS = Performance Status (according to World Health Organization classification),

^a^ no linear relation.

**Fig 3:**
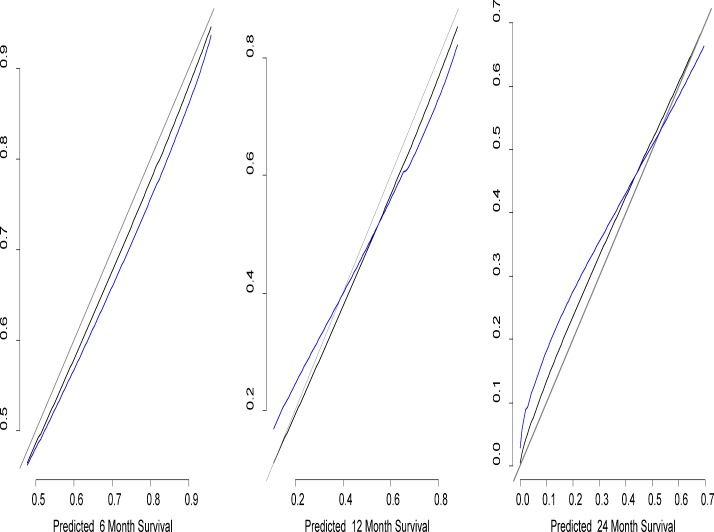
Receiver operating characteristic curves of the calibration. Estimate of calibration accuracy was performed using adaptive spline regression. The line adjacent the ideal line corresponds to the apparent predictive accuracy. The blue line corresponds to corrected estimates.

Although number of organs involved by metastatic disease and type of therapy received (locoregional, systemic or combined) showed some association with prognosis, this was not significant. No association with survival was noted for sex, liver function tests other than LDH and age of metastasis. Remodeling the nomogram to include molecular alterations did not improve its performance. The calibration accuracy was confirmed by the receiver operating characteristic curves shown in Fig. 3. The absence of systematic bias is confirmed by the closeness of the receiver operating characteristic curves; rough shrinkage was 0.9. The nomogram was validated with the external dataset of MC patients by assessing the reliability, as reported in Table 3. The concordance probability was 0.75 (SE. 006) in the development dataset, and 0.80 (SE. 009) in the external validation. S2 table shows the Proportional Hazard Assumption confirmation of the final model. S2 Fig. shows an example of the nomogram use.

**Table 3:** External validation of the nomogram.

Nomogam			External validation		
C Index	Dxy	SE	C Index	Dxy	SE
0.75	0.50	.006	0.80	0.60	.009

SE = Standard Error.

## Discussion

We developed a nomogram that reliably stratifies prognosis for patients with mUM. This nomogram may allow oncologists treating patients with mUM to tailor treatments, allowing for better allocation of resources. Currently, surgeons and oncologists treating patients with mUM lack both a validated system to predict the patient prognosis and reliable decision making tools that may allow for the identification of patients who may actually be harmed by treatment of their metastatic disease. Although this is not as large of a concern in cancers that carry a relative good prognosis and have multiple treatment options with proven clinical benefit, it is a crucial determinant of clinical care for very rare tumors with no established standard treatment and potentially toxic therapies [28].

The two largest previously published series of patients with mUM did evaluate for prognostic factors [3, 34]. Other studies have tried to identify prognostic factors for patients with mUM, however these analyses were based on small series. Moreover, these works took into account only a limited number of putative prognostic factors [3, 5–10, 35, 36]. Of these studies, Eskelin *et*. *al* [6] constructed a prognostic model with PS, dimensions of liver metastasis, ALP levels (as substitute of LDH) and time on treatment using a multivariate analysis in 54 patients. Kodjikian *et al*. found that ciliary body involvement and more than 10 metastases conferred a worse prognosis by analyzing primary melanoma characteristics, age and the number of liver metastasis before surgery in 63 patients using a multivariate Cox regression. Finally, Rietschel *et al*. [10], showed that lung/soft tissue metastases, long DFI, locoregional treatments, female sex and younger age all conferred better prognosis, using a multivariate analysis on 119 patients. Unfortunately, none of these studies considered all of the previously identified prognostic factors and compared them in a multivariate analysis, nor did they adopt any calibration or validation strategy. These previous experiences testify to the difficulty faced when studying the prognostic factors for UM; the rarity of this disease makes the collection of a large, comprehensive series of all prognostic factors complex, with wide variations in diagnostic and treatment modalities over the time of observation. However, with the availability of new regional therapies and targeted drugs, a simple and validated model for patient risk stratification is needed. A reliable tool to evaluate the prognosis could aid clinicians in selecting the candidates for invasive or potentially toxic treatments, which should be reserved for patients with longer life expectancy. With the collaboration of two independent groups, enough data were collected to perform a reliable validation of a prognostic model, with modalities and sample sizes comparable to currently accepted nomograms for rare tumors[36].

Both clinico-pathological and modern molecular prognostic factors have been identified for primary uveal melanoma and are predictors of metastases. However, their usefulness in the metastatic setting has yet to be evaluated. Additionally, the modern molecular procedures, that have been demonstrated to be superior to clinic-pathological characteristics[37], are currently limited to experimental studies. Primary melanoma features, included ciliary body involvement, did not impact on survival in our series. However, we observed a trend for shortest DFI associated to worse primary melanoma characteristics, and this is consistent with the hypothesis that DFI is influenced by the biological aggressiveness of the tumor (reflected by primary melanoma characteristics), but also the result of the interaction of several concurrent variables (such as biological determinants of tumor and immunological equilibrium). We analyzed whether the addition of molecular alterations could improve the performance of the nomogram, however no statistically significant trends with these alterations were noted. One reason for this could be that these alterations have been associated with the development of metastatic disease for patients with mUM, while this study looked at the prognosis of patients who have already developed metastatic disease. The genomic (mainly chromosome 3, 6 and 8 aberrations [38, 39]) and genetic (for example the two gene-expression profiles identified by Onken *et al*. [40]) abnormalities are predictors of distant recurrence after UM primary diagnosis, then other biological factors, yet to be studied, may determine the aggressiveness of metastases when the disease has spread. We encourage future studies to explore the potential genomic and genetic alterations that may influence the prognosis of patients with mUM. Our experience suggests that large, collaborative studies are needed to obtain an adequate sample size to study potential molecular predictors of survival or new therapeutic options, given the rarity of the disease. Melanin is described as one of the potential causes for melanoma refractoriness to treatments[41–44]. There are no extensive data in the literature on the pattern of pigmentation and its prognostic importance in this rare subtype of melanoma. In our series all cases presented strong pigmentation. These data, although insufficient to assert conclusions, are hypothesis generating for further studies focused on mUM, also in the prospective to find the reasons of mUM poorer response to immunotherapies compared to its skin counterpart[13].

In both of our independent series, the survival is longer than other studies; however, similar survival times have been reported by Rietschel *et al*. [10] and Kodjikian *et al*. [45]. It is often not possible to extrapolate the time from the onset of stage IV disease and to that of the initiation of therapy from clinical trials. Additionally, most of the variables we studied were only partially included in other works, making it difficult to compare the OS and clinical predictors of our study with those that have been previously reported. Possible explanations for the differences in survival could include referral bias as both centers included in this study are large referral centers and lead time bias, as we noted shorter DFIs (25.2 and 24.1 months for the IOV and MC dataset, respectively) than those previously reported (30 months in asymptomatic and 79 in symptomatic patients [10]). These differences were unlikely a consequence of the regular liver surveillance, as patients were referred from centers with different follow-up practices, varying from regular liver function tests and liver ultrasound every 6 months to no surveillance. Regardless, the analysis performed was not influenced by the duration of survival itself, but rather by the influence different factors had on survival. To confirm the reproducibility of our results, the nomogram was validated with very good performance despite the significant differences observed for some prognostic predictors between IOV’s and Mayo’s patients. We obtained two bi-phasic survival curves, with a steep slope flatting out after about 20 months (Fig. 1), similar to the curve reported by Rietschel *et al*. [10], suggesting as a confirmation of the heterogeneity of this disease. Although the liver was the most prevalent metastatic site, we identified a number of patients who had metastases in other organs. However, the majority of the patients with mUM died as a result of liver progression despite first developing metastatic disease at another site. Therefore, we cannot advise for routine screening of extra-hepatic sites.

## Conclusions

In summary, we developed and externally validated a nomogram that predicts survival in patients with mUM. This nomogram may be useful in stratifying patients in future clinical trials and help providers prognosticate.

## Supporting Information

S1 Fig The effect of individual predictors on log survival time. A value of zero was used as the reference value for all predicted 95% confidence intervals are shown. “Rug plots” on curves show the density of the predictor.
(TIF)

S2 Fig Simulation of nomogram use. Total points for a patient with PS = 0 (0 points), 20% of liver replacement by metastatic disease (11 points), a LDH serum twice the upper normal limit (72 points), and metastases diagnosed 6 months after initial diagnosis of UM (33 points) were tabulated. The sum 116, corresponds to a survival probability of 0.87 at 6 months, 0.68 at 12 months and approximately 0.35 at 24 months.
(TIF)

S1 table Primary melanoma characteristics, impact on survival and results in multivariate analysis. Primary melanoma characteristics did not have a significant prognostic value in multivariate survival analysis of metastatic patients. 95% CI and *p* refer to the multivariate analysis required to determine the prognostic factors for the model.
(DOC)

S2 table Proportional Hazard confirmation by Schoenfeld residuals. Proportional Hazard hypothesis is confirmed.
(DOCX)

## References

[1] Mallone S, De Vries E, Guzzo M, Midena E, Verne J, Coebergh JW, et al Descriptive epidemiology of malignant mucosal and uveal melanomas and adnexal skin carcinomas in Europe. Eur J Cancer. 2012;48(8):1167–75. 10.1016/j.ejca.2011.10.004 22119735

[2] Diener-West M, Reynolds SM, Agugliaro DJ, Caldwell R, Cumming K, Earle JD, et al Development of metastatic disease after enrollment in the COMS trials for treatment of choroidal melanoma: Collaborative Ocular Melanoma Study Group Report No. 26. Arch Ophthalmol. 2005;123(12):1639–43. 16344433 10.1001/archopht.123.12.1639

[3] Gragoudas ES, Egan KM, Seddon JM, Glynn RJ, Walsh SM, Finn SM, et al Survival of patients with metastases from uveal melanoma. Ophthalmology. 1991;98(3):383–9; discussion 90 2023760 10.1016/s0161-6420(91)32285-1

[4] Augsburger JJ, Correa ZM, Shaikh AH. Effectiveness of treatments for metastatic uveal melanoma. Am J Ophthalmol. 2009;148(1):119–27. 10.1016/j.ajo.2009.01.023 19375060

[5] Rajpal S, Moore R, Karakousis CP. Survival in metastatic ocular melanoma. Cancer. 1983;52(2):334–6. 6190546 10.1002/1097-0142(19830715)52:2<334::aid-cncr2820520225>3.0.co;2-e

[6] Eskelin S, Pyrhonen S, Hahka-Kemppinen M, Tuomaala S, Kivela T. A prognostic model and staging for metastatic uveal melanoma. Cancer. 2003;97(2):465–75. 12518371 10.1002/cncr.11113

[7] Bedikian AY, Kantarjian H, Young SE, Bodey GP. Prognosis in metastatic choroidal melanoma. South Med J. 1981;74(5):574–7. 7244714 10.1097/00007611-198105000-00017

[8] Bedikian AY, Legha SS, Mavligit G, Carrasco CH, Khorana S, Plager C, et al Treatment of uveal melanoma metastatic to the liver: a review of the M. D. Anderson Cancer Center experience and prognostic factors. Cancer. 1995;76(9):1665–70. 8635073 10.1002/1097-0142(19951101)76:9<1665::aid-cncr2820760925>3.0.co;2-j

[9] Hsueh EC, Essner R, Foshag LJ, Ye X, Wang HJ, Morton DL. Prolonged survival after complete resection of metastases from intraocular melanoma. Cancer. 2004;100(1):122–9. 14692032 10.1002/cncr.11872

[10] Rietschel P, Panageas KS, Hanlon C, Patel A, Abramson DH, Chapman PB. Variates of survival in metastatic uveal melanoma. J Clin Oncol. 2005;23(31):8076–80. 16258106 10.1200/JCO.2005.02.6534

[11] Slominski AT, Carlson JA. Melanoma resistance: a bright future for academicians and a challenge for patient advocates. Mayo Clin Proc. 2014;89(4):429–33. 10.1016/j.mayocp.2014.02.009 24684870 PMC4050658

[12] Cruz F 3rd, Rubin BP, Wilson D, Town A, Schroeder A, Haley A, et al Absence of BRAF and NRAS mutations in uveal melanoma. Cancer Res. 2003;63(18):5761–6. 14522897

[13] Maio M, Danielli R, Chiarion-Sileni V, Pigozzo J, Parmiani G, Ridolfi R, et al Efficacy and safety of ipilimumab in patients with pre-treated, uveal melanoma. Ann Oncol. 2013;24(11):2911–5. 10.1093/annonc/mdt376 24067719

[14] Mariani P, Servois V, Piperno-Neumann S. Therapeutic options in metastatic uveal melanoma. Dev Ophthalmol. 2012;49:166–81. 10.1159/000328333 22042020

[15] Peters S, Voelter V, Zografos L, Pampallona S, Popescu R, Gillet M, et al Intra-arterial hepatic fotemustine for the treatment of liver metastases from uveal melanoma: experience in 101 patients. Ann Oncol. 2006;17(4):578–83. 16469752 10.1093/annonc/mdl009

[16] Leyvraz S, Piperno-Neumann S, Suciu S, Baurain JF, Zdzienicki M, Testori A, et al Hepatic intra-arterial versus intravenous fotemustine in patients with liver metastases from uveal melanoma (EORTC 18021): a multicentric randomized trial. Ann Oncol. 2014;25(3):742–6. 10.1093/annonc/mdt585 24510314 PMC4433517

[17] Sato T, Eschelman DJ, Gonsalves CF, Terai M, Chervoneva I, McCue PA, et al Immunoembolization of malignant liver tumors, including uveal melanoma, using granulocyte-macrophage colony-stimulating factor. J Clin Oncol. 2008;26(33):5436–42. 10.1200/JCO.2008.16.0705 18838710 PMC6815970

[18] Fiorentini G, Aliberti C, Del Conte A, Tilli M, Rossi S, Ballardini P, et al Intra-arterial hepatic chemoembolization (TACE) of liver metastases from ocular melanoma with slow-release irinotecan-eluting beads. Early results of a phase II clinical study. In Vivo. 2009;23(1):131–7. 19368137

[19] Gonsalves CF, Eschelman DJ, Sullivan KL, Anne PR, Doyle L, Sato T. Radioembolization as salvage therapy for hepatic metastasis of uveal melanoma: a single-institution experience. AJR Am J Roentgenol. 2011;196(2):468–73. 10.2214/AJR.10.4881 21257902

[20] Feldman ED, Pingpank JF, Alexander HR Jr. Regional treatment options for patients with ocular melanoma metastatic to the liver. Ann Surg Oncol. 2004;11(3):290–7. 14993024 10.1245/aso.2004.07.004

[21] Olofsson R, Cahlin C, All-Ericsson C, Hashimi F, Mattsson J, Rizell M, et al Isolated Hepatic Perfusion for Ocular Melanoma Metastasis: Registry Data Suggests a Survival Benefit. Ann Surg Oncol. 2013. 10.1245/s10434-013-3304-z 24141377

[22] Leyvraz S, Keilholz U. Ocular melanoma: what's new? Curr Opin Oncol. 2012;24(2):162–9. 10.1097/CCO.0b013e32834ff069 22234256

[23] Kivela T, Suciu S, Hansson J, Kruit WH, Vuoristo MS, Kloke O, et al Bleomycin, vincristine, lomustine and dacarbazine (BOLD) in combination with recombinant interferon alpha-2b for metastatic uveal melanoma. Eur J Cancer. 2003;39(8):1115–20. 12736111 10.1016/s0959-8049(03)00132-1

[24] Dorval T, Fridman WH, Mathiot C, Pouillart P. Interleukin-2 therapy for metastatic uveal melanoma. Eur J Cancer. 1992;28A(12):2087 1419309 10.1016/0959-8049(92)90266-5

[25] Valsecchi ME, Sato T. The potential role of sunitinib targeting melanomas. Expert Opin Investig Drugs. 2013;22(11):1473–83. 10.1517/13543784.2013.837449 24050392

[26] Tarhini AA, Frankel P, Margolin KA, Christensen S, Ruel C, Shipe-Spotloe J, et al Aflibercept (VEGF Trap) in inoperable stage III or stage iv melanoma of cutaneous or uveal origin. Clin Cancer Res. 2011;17(20):6574–81. 10.1158/1078-0432.CCR-11-1463 21880788 PMC3196047

[27] Falchook GS, Lewis KD, Infante JR, Gordon MS, Vogelzang NJ, DeMarini DJ, et al Activity of the oral MEK inhibitor trametinib in patients with advanced melanoma: a phase 1 dose-escalation trial. Lancet Oncol. 2012;13(8):782–9. 10.1016/S1470-2045(12)70269-3 22805292 PMC4109286

[28] Carvajal RD, Sosman JA, Quevedo JF, Milhem MM, Joshua AM, Kudchadkar RR, et al Effect of selumetinib vs chemotherapy on progression-free survival in uveal melanoma: a randomized clinical trial. JAMA. 2014;311(23):2397–405. 10.1001/jama.2014.6096 24938562 PMC4249701

[29] Midena E, Bonaldi L, Parrozzani R, Tebaldi E, Boccassini B, Vujosevic S. In vivo detection of monosomy 3 in eyes with medium-sized uveal melanoma using transscleral fine needle aspiration biopsy. Eur J Ophthalmol. 2006;16(3):422–5. 16761244 10.1177/112067210601600310

[30] Midena E, Bonaldi L, Parrozzani R, Radin PP, Boccassini B, Vujosevic S. In vivo monosomy 3 detection of posterior uveal melanoma: 3-year follow-up. Graefes Arch Clin Exp Ophthalmol. 2008;246(4):609–14. 17934749 10.1007/s00417-007-0692-4

[31] Bonaldi L, Midena E, Filippi B, Tebaldi E, Marcato R, Parrozzani R, et al FISH analysis of chromosomes 3 and 6 on fine needle aspiration biopsy samples identifies distinct subgroups of uveal melanomas. J Cancer Res Clin Oncol. 2008;134(10):1123–7. 10.1007/s00432-008-0382-6 18386059 PMC12161757

[32] Valpione S, Aliberti C, Parrozzani R, Bazzi M, Pigozzo J, Midena E, et al A retrospective analysis of 141 patients with liver metastases from uveal melanoma: a two-cohort study comparing transarterial chemoembolization with CPT-11 charged microbeads and historical treatments. Melanoma Res. 2015. 10.1097/CMR.0000000000000129 25521594

[33] Moser JC, Pulido JS, Dronca RS, McWilliams RR, Markovic SN, Mansfield AS. The Mayo Clinic experience with the use of kinase inhibitors, ipilimumab, bevacizumab, and local therapies in the treatment of metastatic uveal melanoma. Melanoma Res. 2015;25(1):59–63. 10.1097/CMR.0000000000000125 25396683

[34] Diener-West M, Reynolds SM, Agugliaro DJ, Caldwell R, Cumming K, Earle JD, et al Screening for metastasis from choroidal melanoma: the Collaborative Ocular Melanoma Study Group Report 23. J Clin Oncol. 2004;22(12):2438–44. 15197206 10.1200/JCO.2004.08.194

[35] Kath R, Hayungs J, Bornfeld N, Sauerwein W, Hoffken K, Seeber S. Prognosis and treatment of disseminated uveal melanoma. Cancer. 1993;72(7):2219–23. 7848381 10.1002/1097-0142(19931001)72:7<2219::aid-cncr2820720725>3.0.co;2-j

[36] Gold JS, Gonen M, Gutierrez A, Broto JM, Garcia-del-Muro X, Smyrk TC, et al Development and validation of a prognostic nomogram for recurrence-free survival after complete surgical resection of localised primary gastrointestinal stromal tumour: a retrospective analysis. Lancet Oncol. 2009;10(11):1045–52. 10.1016/S1470-2045(09)70242-6 19793678 PMC3175638

[37] Augsburger JJ, Correa ZM, Trichopoulos N. An alternative hypothesis for observed mortality rates due to metastasis after treatment of choroidal melanomas of different sizes. Trans Am Ophthalmol Soc. 2007;105:54–9; discussion 9–60 18427594 PMC2258111

[38] Ewens KG, Kanetsky PA, Richards-Yutz J, Al-Dahmash S, De Luca MC, Bianciotto CG, et al Genomic profile of 320 uveal melanoma cases: chromosome 8p-loss and metastatic outcome. Invest Ophthalmol Vis Sci. 2013;54(8):5721–9. 10.1167/iovs.13-12195 23821189

[39] Thomas S, Putter C, Weber S, Bornfeld N, Lohmann DR, Zeschnigk M. Prognostic significance of chromosome 3 alterations determined by microsatellite analysis in uveal melanoma: a long-term follow-up study. Br J Cancer. 2012;106(6):1171–6. 10.1038/bjc.2012.54 22353812 PMC3304422

[40] Onken MD, Worley LA, Ehlers JP, Harbour JW. Gene expression profiling in uveal melanoma reveals two molecular classes and predicts metastatic death. Cancer Res. 2004;64(20):7205–9. 15492234 10.1158/0008-5472.CAN-04-1750 PMC5407684

[41] Brozyna AA, VanMiddlesworth L, Slominski AT. Inhibition of melanogenesis as a radiation sensitizer for melanoma therapy. Int J Cancer. 2008;123(6):1448–56. 10.1002/ijc.23664 18567001

[42] Slominski A, Zbytek B, Slominski R. Inhibitors of melanogenesis increase toxicity of cyclophosphamide and lymphocytes against melanoma cells. Int J Cancer. 2009;124(6):1470–7. 10.1002/ijc.24005 19085934 PMC2628959

[43] Slominski A, Zmijewski MA, Pawelek J. L-tyrosine and L-dihydroxyphenylalanine as hormone-like regulators of melanocyte functions. Pigment Cell Melanoma Res. 2012;25(1):14–27. 10.1111/j.1755-148X.2011.00898.x 21834848 PMC3242935

[44] Brozyna AA, Jozwicki W, Carlson JA, Slominski AT. Melanogenesis affects overall and disease-free survival in patients with stage III and IV melanoma. Hum Pathol. 2013;44(10):2071–4. 10.1016/j.humpath.2013.02.022 23791398 PMC3783651

[45] Kodjikian L, Grange JD, Baldo S, Baillif S, Garweg JG, Rivoire M. Prognostic factors of liver metastases from uveal melanoma. Graefes Arch Clin Exp Ophthalmol. 2005;243(10):985–93. 15891893 10.1007/s00417-005-1188-8

