# Supplementary figures and images for: Development and External Validation of a Prognostic Nomogram for Metastatic Uveal Melanoma
Source: PLoS One. 2015 Mar 17;10(3):e0120181. doi: 10.1371/journal.pone.0120181 (PMC4363319; doi:10.1371/journal.pone.0120181)

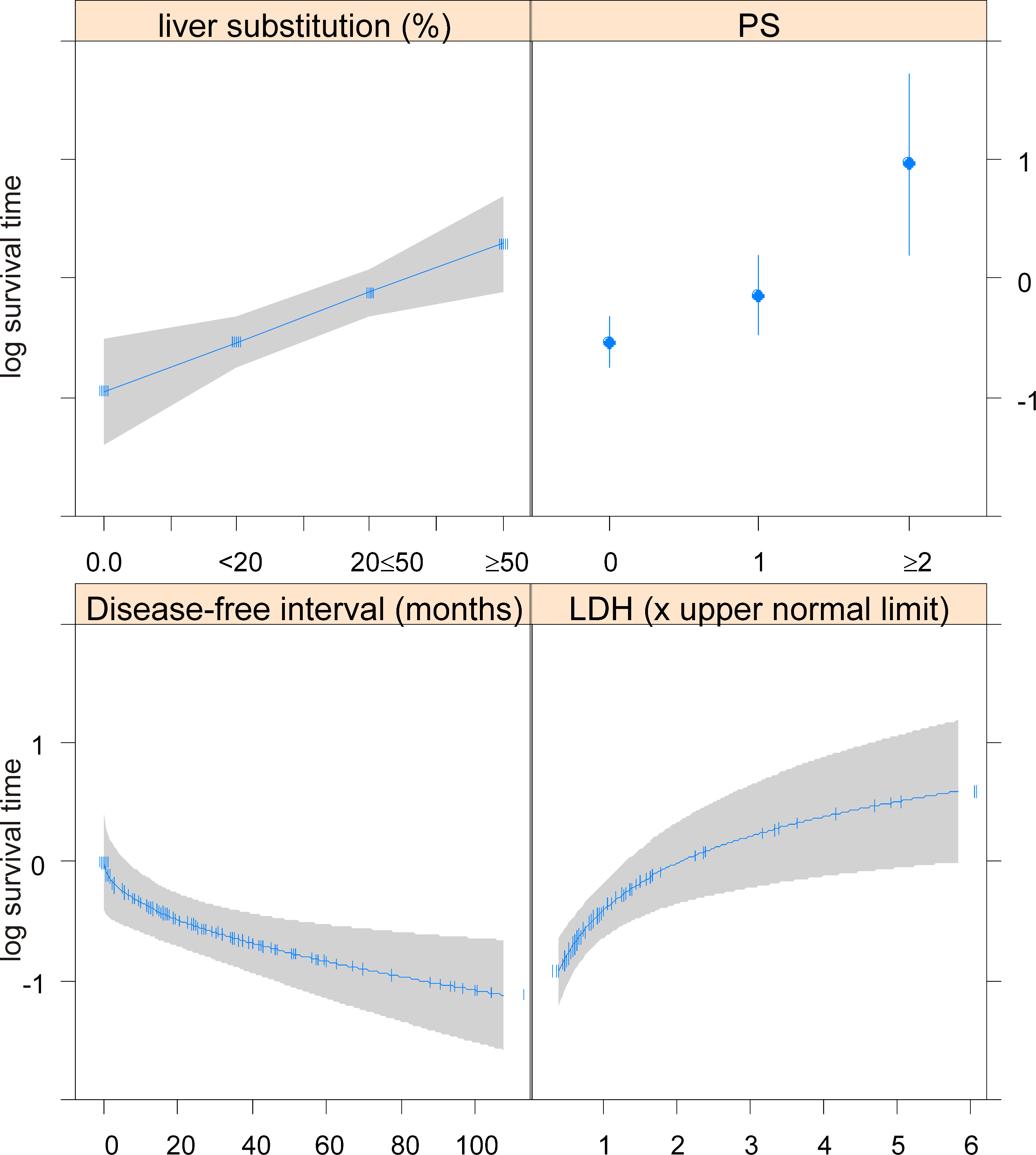

Supplement: S1 Fig — A value of zero was used as the reference value for all predicted 95% confidence intervals are shown. “Rug plots” on curves show the density of the predictor. (TIF) [file pone.0120181.s001.tif]

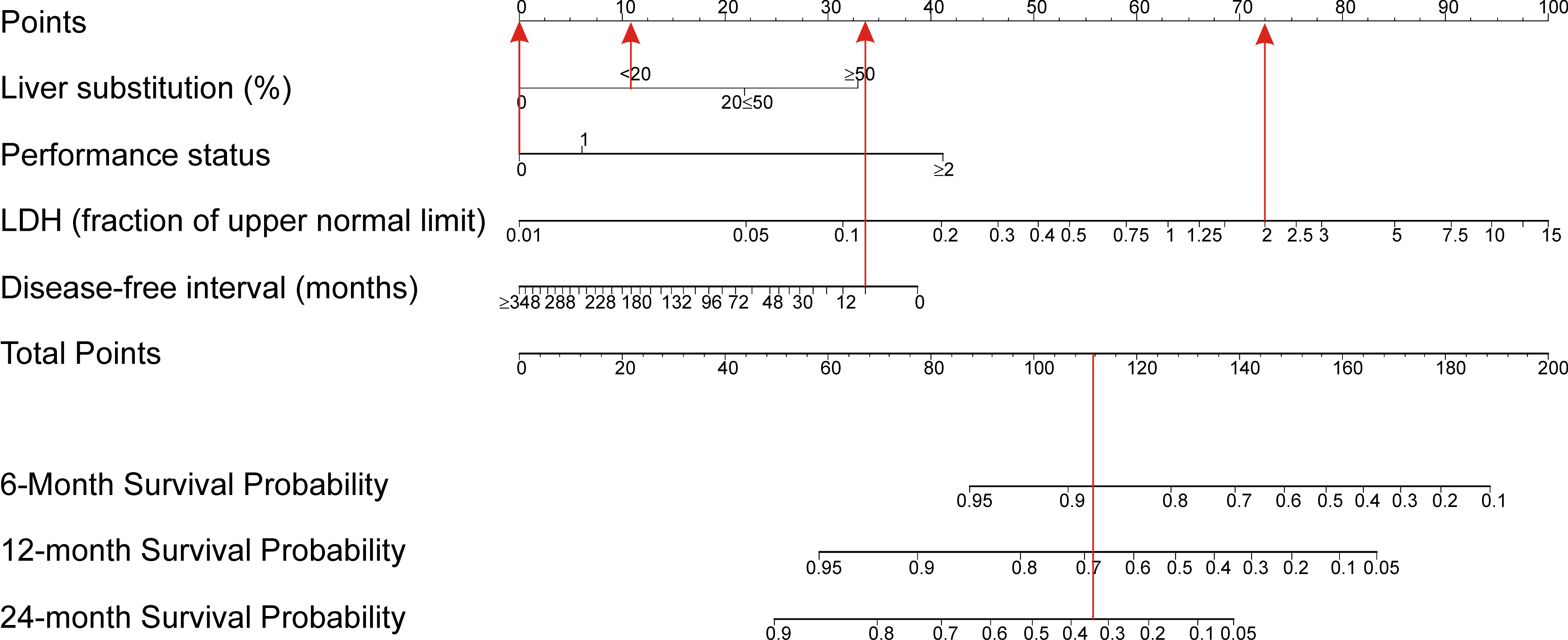

Supplement: S2 Fig — Total points for a patient with PS = 0 (0 points), 20% of liver replacement by metastatic disease (11 points), a LDH serum twice the upper normal limit (72 points), and metastases diagnosed 6 months after initial diagnosis of UM (33 points) were tabulated. The sum 116, corresponds to a survival probability of 0.87 at 6 months, 0.68 at 12 months and approximately 0.35 at 24 months. (TIF) [file pone.0120181.s002.tif]
