# Supplementary material for: Development and External Validation of a Prognostic Nomogram for Metastatic Uveal Melanoma
Source: PLoS One. 2015 Mar 17;10(3):e0120181. doi: 10.1371/journal.pone.0120181 (PMC4363319; doi:10.1371/journal.pone.0120181)
Supplement: S1 table — Primary melanoma characteristics did not have a significant prognostic value in multivariate survival analysis of metastatic patients. 95% CI and p refer to the multivariate analysis required to determine the prognostic factors for the model. (DOC) [file pone.0120181.s003.doc]

**SUPPLEMENTARY TABLES**

Table S1 Primary melanoma characteristics, impact on survival and results in multivariate analysis

| Variable | Number or Median | % | Median survival (months) | HR | 95% CI | *p* |
| --- | --- | --- | --- | --- | --- | --- |
| Stage |  |  |  |  |  |  |
| T1 | 29 | 19 | 25.9 | 0.71 | 0.01-3.80 | .256 |
| T2 | 50 | 33 | 14.0 | 0.33 | 0.03-3.91 | .380 |
| T3 | 65 | 43 | 12.7 | 0.30 | 0.05-1.89 | .202 |
| T4 | 8 | 5 | 5.1 | 1 | - | - |
| Thickness (mm) | 6.1 |  | 17.2 | 1.07 | 0.91-1.27 | .463 |
| Larger Base Diameter (mm) | 13 |  | 17.2 | 0.92 | 0.80-1.05 | .396 |
| Localization |  |  |  |  |  |  |
| Ciliary body involvement | 11 | 7 | 19.9 | 0.71 | 0.35-1.44 | .342 |
| Choroidal | 141 | 93 | 15.4 | 1 |  |  |
| Histologic subtype^a^ |  |  |  |  |  |  |
| Epitheloid | 3 | 2 | 15.6 | 4.30 | 0.01-100 | .981 |
| Mixed | 146 | 96 | 16.8 | 1.52 | 0.26-1.64 | .125 |
| Spindle cell | 3 | 2 | 18.1 | 1 |  |  |
| Pigmentation^b^ |  |  |  |  |  |  |
| Amelanotic (score=0) | 0 | 0 | - | - | - | - |
| Moderately pigmented (score=1 or 2) | 0 | 0 | - | - | - | - |
| Strongly pigmented (score=3) | 150 | 100 | 17.2 | - | - | - |
| *Not evaluable* | 2 |  |  |  |  |  |
| Chromosomal alterations |  |  |  |  |  |  |
| None | 16 | 16 | 16.5 | 1 | - | - |
| ..Chromosome 3alteration |  |  |  |  |  |  |
| loss | 75 | 75 | 17.6 | 0.87 | 0.57-1.13 | .517 |
| trisomy | 1 | 1 | 14.6 | 3.12 | 0.01-100 | .981 |
| quadrisomy | 1 | 1 | 7.5 | 5.77 | 0.01-100 | .981 |
| Chromosome 8 gain | 1 | 1 | 24.8 | 0.38 | 0.01-100 | .981 |
| Chromosome 6 gain | 0 | - | - | - | - | - |
| Chromosome 10 alteration |  |  |  |  |  |  |
| trisomy | 2 | 2 | 11.9 | 3.25 | 0.01-100 | .981 |
| quadrisomy | 1 | 1 | 15.3 | 1.71 | 0.01-100 | .981 |
| Multiple alterations | 2 | 2 | 4.8 | 32.0 | 0.01-100 | .981 |
| Aneuploidy | 1 | 1 | 16.2 | 1.14 | 0.01-100 | .981 |
| *Not evaluable* | 50 |  |  |  |  |  |

^a^ primary melanoma was enucleated or confirmed by fine needle biopsy of primary tumor;

^b^ see Brozyna et al. 2012, for detailed description of the pigmentation score
