# Supplementary material for: Development and External Validation of a Prognostic Nomogram for Metastatic Uveal Melanoma
Source: PLoS One. 2015 Mar 17;10(3):e0120181. doi: 10.1371/journal.pone.0120181 (PMC4363319; doi:10.1371/journal.pone.0120181)
Supplement: S2 table — Proportional Hazard hypothesis is confirmed. (DOCX) [file pone.0120181.s004.docx]

**SUPPLEMENTARY TABLES**

Table S2 Proportional Hazard confirmation by Schoenfeld residuals

| Covariate | rho | chi2 | *p* |
| --- | --- | --- | --- |
| liver_substitution | -8,77 | 13,69 | .274 |
| PS | -0,43 | 0,26 | .951 |
| LDH | -3,81 | 18,22 | .608 |
| free_interval | -4,84 | 28,69 | .520 |
| GLOBAL | 0 | 11,54 | .705 |
